# Functional Comparison of Human Adenomatous Polyposis Coli (APC) and APC-Like in Targeting Beta-Catenin for Degradation

**DOI:** 10.1371/journal.pone.0068072

**Published:** 2013-07-01

**Authors:** Jean Schneikert, Shree Harsha Vijaya Chandra, Jan Gustav Ruppert, Suparna Ray, Eva Maria Wenzel, Jürgen Behrens

**Affiliations:** 1 Nikolaus Fiebiger Center for Molecular Medicine, University of Erlangen-Nürnberg, Erlangen, Germany; 2 Department of Biochemistry, Institute for Cancer Research, The Norwegian Radium Hospital, Oslo University Hospital and Centre for Cancer Biomedicine, Faculty of Medicine, University of Oslo, Oslo, Norway; University of Munich, Germany

## Abstract

Truncating mutations affect the adenomatous polyposis coli (APC) gene in most cases of colon cancer, resulting in the stabilization of β-catenin and uncontrolled cell proliferation. We show here that colon cancer cell lines express also the paralog APC-like (APCL or APC2). RNA interference revealed that it controls the level and/or the activity of β-catenin, but it is less efficient and binds less well to β-catenin than APC, thereby providing one explanation as to why the gene is not mutated in colon cancer. A further comparison indicates that APCL down-regulates the β-catenin level despite the lack of the 15R region known to be important in APC. To understand this discrepancy, we performed immunoprecipitation experiments that revealed that phosphorylated β-catenin displays a preference for binding to the 15 amino acid repeats (15R) rather than the first 20 amino acid repeat of APC. This suggests that the 15R region constitutes a gate connecting the steps of β-catenin phosphorylation and subsequent ubiquitination/degradation. Using RNA interference and domain swapping experiments, we show that APCL benefits from the 15R of truncated APC to target β-catenin for degradation, in a process likely involving heterodimerization of the two partners. Our data suggest that the functional complementation of APCL by APC constitutes a substantial facet of tumour development, because the truncating mutations of APC in colorectal tumours from familial adenomatous polyposis (FAP) patients are almost always selected for the retention of at least one 15R.

## Introduction

The APC gene [1] is mutated in the vast majority of colon cancers [2] and a significant proportion of other tumours [3]–[7]. The APC protein displays many different functions [8]–[10]. It is best known, together with axin/axin2 and the kinases glycogen synthase kinase 3 beta (GSK3β) and casein kinase 1 alpha (CK1α), as a component of the destruction complex that initiates the phosphorylation-dependent degradation of β-catenin [11]. β-catenin is a transcription factor [12], the main effector of the wnt signaling pathway that stimulates the proliferation of the few stem cells located at the bottom of the crypts of the colonic epithelium [13]. The accumulation of β-catenin in stem cells is tightly regulated by the destruction complex. Mutations of the APC gene in colon cancer result in the translation of products that lack roughly the C-terminal half [14]. The consequences are the deletion of domains involved in β-catenin degradation, the constitutive stabilization of β-catenin and the pathological expansion of the stem cell compartment [13].

**Figure 1:**
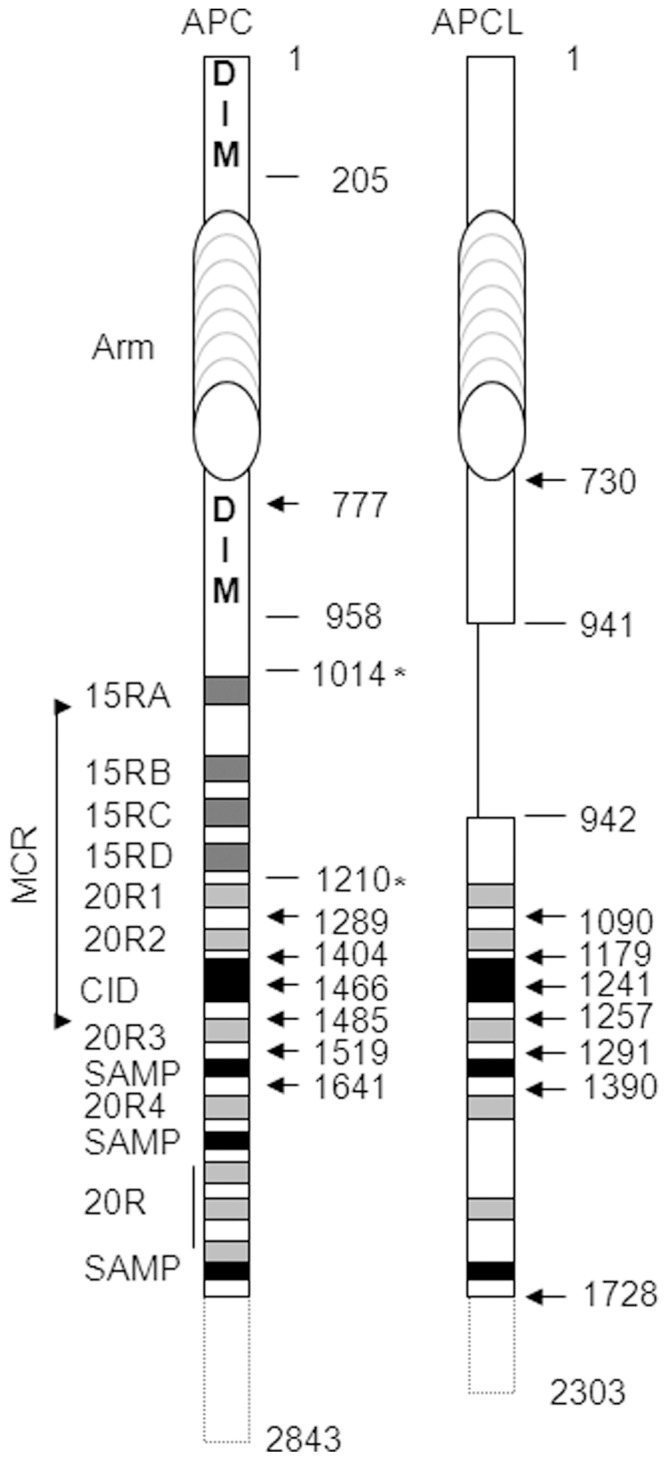
Schematic representation of APC and APCL (total lengths of 2843 and 2303 amino acid residues, respectively). Functional modules are shown, including the two dimerization domains of APC (DIM), the armadillo repeat domain (Arm), the 15 (15RA to D) and 20 (20R1 to7) amino acid repeats that are β-catenin binding sites, the β-catenin inhibitory domain (CiD) involved in β-catenin degradation and the SAMP repeats that are axin/axin2 binding sites. Note that APCL does not contain the 15R region and the second dimerization domain is not conserved. The mutation cluster region (MCR) is the region in which most truncating mutations of APC have been found in colon cancer. Numberings indicate amino acid positions. Those with arrows correspond to the size (from amino acid 1) of the different constructs analyzed in this study and fused to either YFP or RFP at their N-terminus. The amino acid positions of APC denoted with asterisks highlight the borders of the 15R region that has been introduced between residues 941 and 942 of APCL.

Truncating mutations of APC have the tendency to concentrate in the middle of the open reading frame, the so-called mutation cluster region (MCR) [15], [16] (**figure 1**). At the protein level, the C-terminal border of the MCR is located at the beginning of the third 20 amino acid repeat (20R3) [17]. A negative selection precludes, in the vast majority of cases, the presence of the 20R3 which is a high affinity binding site for β-catenin [18]. On the other end, the N-terminal border is located shortly after the first 15 amino acid repeat (15RA). The analysis of the distribution of truncating mutations across the MCR in tumours from patients with the dominantly inherited familial adenomatous polyposis (FAP) syndrome revealed that truncations are almost always selected for the presence of at least the 15RA (in 326 out of 328 tumours) [19], [20]. This suggests that the 15RA plays an important role in tumour development.

Compared to APC, human APCL (or APC2) [21], [22] lacks the C-terminus and the internal region containing the 15 amino acid repeats (15Rs) (**figure 1**). Besides these differences the topological organizations of APC and APCL are conserved. The N-terminal domain of APC mediates homodimerization [23] and it is conserved in APCL. Homodimerization of APCL through the N-terminal domain remains to be demonstrated, and recent data suggest that the Drosophila counterpart E-APC oligomerizes through the armadillo repeat domain [24]. APC contains a second dimerization domain [20], [25], located between the armadillo repeat and the 15R region, but it is not conserved in APCL. APC and APCL can heterodimerize [26], but it is not known whether this involves the N-terminal dimerization domain. Rather, the armadillo repeats seem sufficient [27]. APCL may bind to β-catenin through its 20Rs, but experimental evidence is still lacking. APC and APCL display a conserved β-catenin inhibitory domain (CiD) which is important in APC to target β-catenin for degradation [28]. It is not known whether the CiD of APCL is operational but the orthologous sequence in E-APC from Drosophila is crucial for efficient β-catenin degradation [29]. Finally, both APC and APCL harbour binding sites for axin/axin2 (the so-called SAMP repeats, three in APC [30] versus two in APCL [22]. APCL has been shown to target β-catenin for destruction in cell culture [21], [22], but an APCL knock-out in the mouse brain is not accompanied by the stabilization of β-catenin [31]. However, D-APC and E-APC from Drosophila have redundant functions and compensate each other [32]. APCL mutations have not been reported in tumours of the gastrointestinal tract. We undertook here a functional comparison of APC and APCL.

## Results

### APCL is Expressed in various Colorectal Cancer Cell Lines

**Figure 2:**
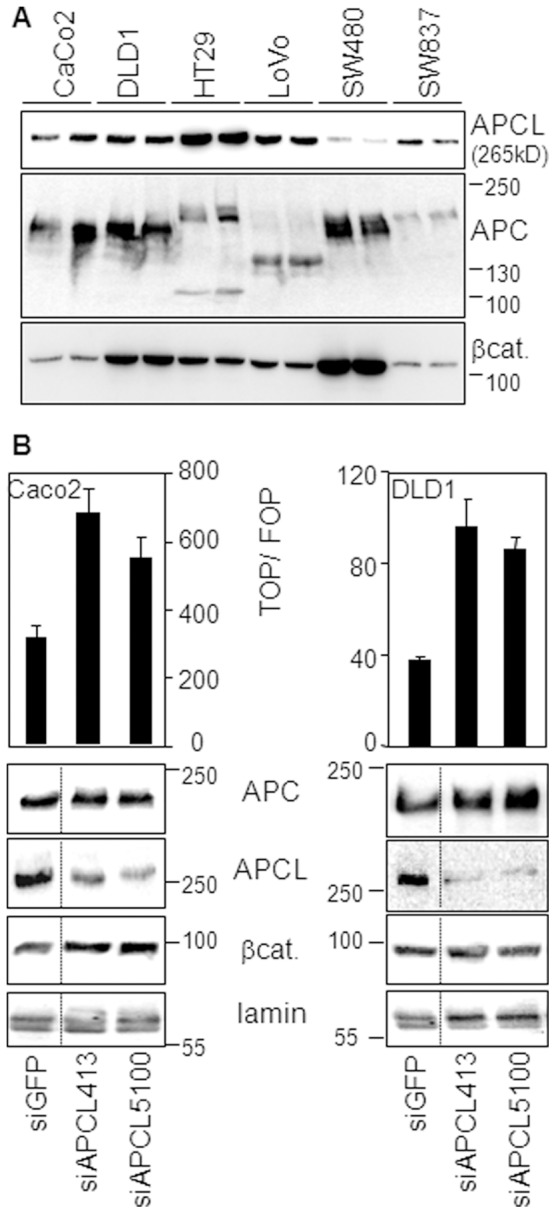
Expression of APCL in colon cancer cell lines and functional consequences after down-regulation by RNA interference. **A,** APCL is expressed in CaCo2, DLD1, HT29, LoVo, SW480 and SW837 colorectal cancer cell lines. Hypotonic cell extracts from cells grown to approximately the same confluence were subjected in duplicate to western blotting analysis, using the indicated antibodies. See material and methods to retrieve a description of the different truncating APC mutations in the cell lines. **B,** APCL down-regulation increases the level of β-catenin in CaCo2 cells and stimulates its transcriptional activity in CaCo2 and DLD1 cells. Cells were transiently transfected with siRNAs to GFP, APC and APCL on day 1 and with reporter plasmids on day 2. TOP/FOP reporter assays were performed on day 4 to measure the transcriptional activity of β-catenin (see Material and Methods). Shown is the mean of three independent values +/− standard deviation from a representative experiment. Hypotonic cell extracts were prepared on day 4 from cells not transfected with plasmids and subjected to western blotting analysis, using the indicated antibodies. Molecular weights are shown in kD. The dotted lines indicate the removal of intervening lanes.

It has been mentioned that the APCL mRNA is expressed in the colon and small intestine [26] and the protein in T84, LS123 and SW480 colorectal cancer cell lines [26]. To extend these observations, we performed an immunoblot analysis using cell extracts from CaCo2, DLD1, HT29, LoVo, SW480 and SW837 colon cancer cells (**figure 2A**). The anti-APCL antibody revealed a single band of the predicted size (265 kD) in all cell lines tested. This band was specific for APCL because RNA interference reduced its intensity (see below). Thus, the APCL protein is present in all colorectal cancer cell lines that have been tested so far. The blots were also probed with anti-APC and anti-β-catenin antibodies, but we did not establish any correlation between the levels of expression of APC, APCL and β-catenin.

### Endogenous APCL Regulates the Activity and/or the Level of β-catenin in Colon Cancer Cells

To investigate the possibility that endogenous APCL may regulate β-catenin activity and/or expression level, two siRNAs against APCL were selected for their ability to down-regulate specifically the expression of APCL but not APC (**figure 2B**). We performed our experiments in CaCo2 and DLD1 cells, in which the transcriptional activity of β-catenin can be easily measured using the TOP/FOP reporter assay (see Methods). When transiently transfected into CaCo2 cells, the siAPCLs led to the down-regulation of APCL, but not APC (**figure 2B, left panel**). The reductions in APCL protein levels were accompanied by an increase in β-catenin levels and transcriptional activity, thereby indicating that APCL regulates the level of β-catenin in Caco2 cells. In DLD1 cells, β-catenin levels were not affected by downregulation of APCL, but the transcriptional activity still increased (**figure 2B, right panel**). Altogether, we concluded that APCL regulates the activity of β-catenin in DLD1 cells and both the activity and the level of β-catenin in CaCo2 cells. The same observations were made previously in these cell lines upon down-regulation of truncated APC [33], [34], and discussed [33]. Of note, down-regulation of APCL in SW480 cells altered neither the β-catenin stady-state, nor its transcriptional activity (unpublished data). This is possibly due to the fact that SW480 cells contain already low levels of APCL as compared to the other colorectal cancer cell lines we analysed (**figure 2A**).

### APCL is Less Efficient than APC in Down-regulating β-catenin Level and Transcriptional Activity

**Figure 3:**
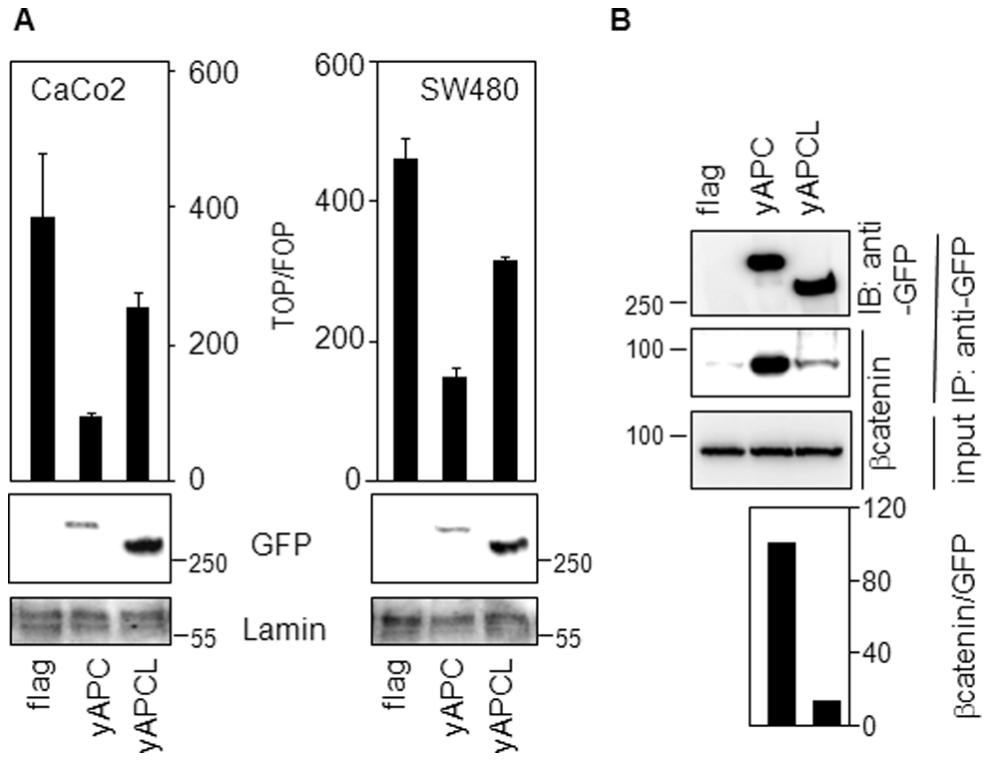
Comparison of yAPC and yAPCL in binding to β-catenin and inhibiting its transcriptional activity. **A**, APCL is less efficient than APC in inhibiting the transcriptional activity of β-catenin. CaCo2 and SW480 cells were transiently transfected on day 1 with reporter plasmids, together with 100 ng of either an empty vector (flag) or expression vectors for either APC or APCL tagged with YFP at their N-terminus. TOP/FOP reporter assays were performed on day 3 to measure the transcriptional activity of β-catenin (see Material and Methods). Shown is the mean of three independent values +/− standard deviation from a representative experiment. In a parallel experiment, cells were transiently transfected with 1 µg of the indicated plasmids on day 1. Whole cell extracts were prepared on day 3 and subjected to western blotting analysis using the indicated antibodies. **B**, APCL binds less well to β-catenin than APC. 293T cells were transiently transfected on day 1 with either a control vector (flag) or expression plasmids for either APC or APCL fused to YFP at their N-terminus. Triton-X100 cell extracts were prepared on day 3, mixed with an SW480 Triton-X100 extract as a source of β-catenin and subjected to immunoprecipitation using an anti-GFP antibody (IP GFP). Pulled-down proteins were revealed by western blotting analysis using the indicated antibodies. The diagram below shows the ratio of the β-catenin signal to the GFP signal obtained after densitometric analysis of the immunoprecipitated products. Molecular weights are shown in kD.

To make a functional comparison of APCL with APC, we constructed an expression vector encoding an N-terminally tagged YFP-APCL (yAPCL) fusion protein (**figure 1**). After transient transfection of yAPCL in SW480 cells in parallel with a YFP-APC (yAPC) fusion construct (**figure 1**) [20], we observed that the intracellular localizations of yAPC and yAPCL differed significantly (**figure S1A**). yAPC displayed a fiber-like appearance, likely reflecting localization at microtubules, as previously characterized [9], [10]. yAPCL concentrated predominantly as dots surrounding the nucleus, which is in agreement with its previously described colocalization with the Golgi apparatus [26]. Additionally, yAPCL also had a fiber-like appearance in SW480 cells (unpublished data), reflecting likely its colocalization with microtubules and/or actin fibers, as previously described for endogenous APCL [26]. Using an antibody against β-catenin, we confirmed that yAPCL, like yAPC, can down-regulate the β-catenin level in SW480 cells that contain sufficient β-catenin to be easily detectable by immunofluorescence (**figure S1A**). When we measured the transcriptional activity of β-catenin in CaCo2 cells using the TOP/FOP luciferase reporter assay (see Methods), we found that yAPCL displays a weaker ability to downregulate β-catenin-dependent transcription than yAPC despite higher expression levels (**figure 3A**). Lowering the amount of yAPCL resulted in an even weaker activity, thereby excluding the possibility of a dominant negative effect linked to high expression level of yAPCL (**figure S1B**). In SW480 cells, yAPCL was also less active than yAPC in inhibiting the transcriptional activity of β-catenin (**figure 3A**). When we compared their β-catenin-binding capacity by immunoprecipitation, we found that yAPCL was less efficient than yAPC in pulling down β-catenin (**figure 3B**). Together, we concluded that APCL can down-regulate the level of β-catenin, but with a weaker efficiency than APC, possibly due to a weaker ability to bind β-catenin and/or a different intracellular localization.

### APCL, like APC, Requires the CiD Domain to Down-regulate the Level of β-catenin

**Figure 4:**
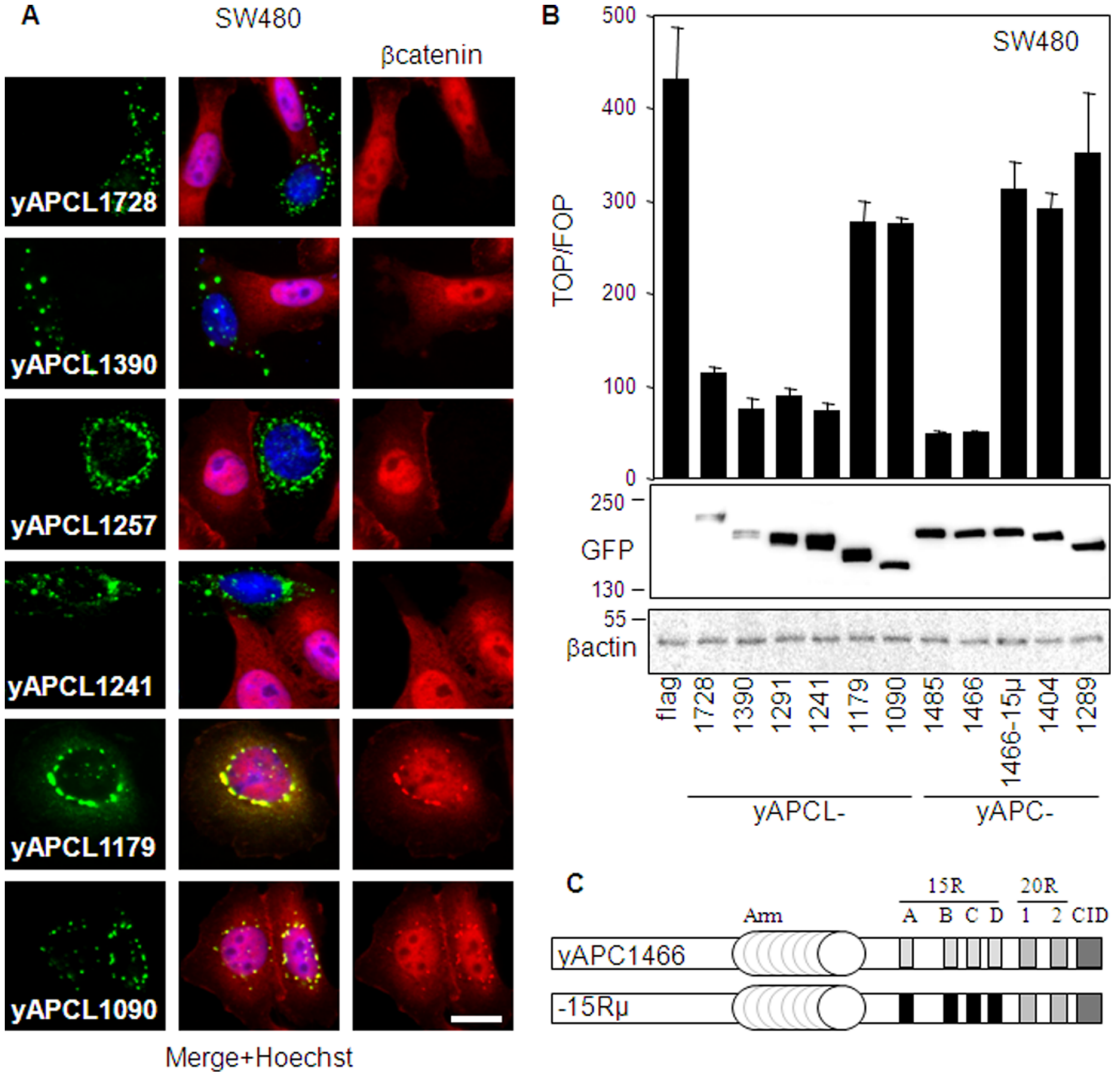
APCL requires the CiD domain to catalyze β-catenin degradation and inhibits its transcriptional activity despite the absence of the 15R region. **A**, APCL requires the CiD domain to catalyze β-catenin degradation. SW480 cells were transiently transfected on day 1 with 1 µg of the indicated APCL constructs tagged with YFP at their N-terminus. Cells were fixed on day 3 and stained with an antibody against β-catenin and the Hoechst dye. The green, red and blue colours correspond to the fluorescences associated with YFP, β-catenin and the Hoechst dye, respectively. Bar, 10 µm. **B**, APCL inhibits the transcriptional activity of β-catenin despite the absence of the 15R region. SW480 cells were transiently transfected on day 1 with reporter plasmids together with 100 ng of either an empty plasmid (flag) or expression vectors for the indicated APCL constructs tagged with YFP at their N-terminus (figure 1). TOP/FOP reporter assays were performed on day 3 to measure the transcriptional activity of β-catenin (see Methods). Shown is the mean of three independent values +/− standard deviation from a representative experiment. In parallel, cells were transiently transfected on day 1 with 1 µg of the indicated constructs and whole cell extracts were prepared on day 3 and subjected to western blotting analysis using the indicated antibodies. Molecular weights are shown in kD. **C,** Schematic representation of yAPC1466 and yAPC1466-15Rµ. See figure 1 for the nomenclature. The repeats indicated in black are mutated and cannot bind to β-catenin.

In the next step, we used yAPCL deletion constructs (**figure 1**) in immunofluorescence experiments to define the shortest fragment sufficient to down-regulate the level of β-catenin in SW480 cells (**figure 4A**). The intracellular localizations of all constructs were very similar and consisted in bright punctuate structures surrounding the nucleus and exclusively cytoplasmic (**figure S2**), which is again in agreement with the previously described localization of APCL to the Golgi apparatus [26]. We found that yAPCL1241 and larger constructs (yAPCL1257, yAPCL1390 and yAPCL1728) were decreasing the level of β-catenin in SW480 cells, whereas yAPCL1179 and yAPCL1090 were inactive (**figure 4A**). Thus, shortening yAPCL1241 down to yAPCL1179 abrogates down-regulation of the β-catenin level. The region spanning amino acid residues 1179 to 1241 of APCL is highly homologous to the CiD domain of APC [28], or B domain in Drosophila [29], which have been previously characterized as important determinants of β-catenin destruction.

### Truncated APCL does not Require the 15R to Inhibit the Transcriptional Activity of β-catenin

We compared the relative efficacies of the various yAPCL deletion constructs with those of their corresponding yAPC truncations in regulating β-catenin-dependent transcription in TOP/FOP reporter assays (**figure 4B**). We found that the inhibition of the transcriptional activity of β-catenin by yAPCL and yAPC was dramatically reduced as soon as their CiD domains were deleted, thereby strengthening the notion that yAPCL and yAPC share the same mechanism of β-catenin destruction involving the CiD domains. However, a major difference was observed. The 15Rs were important to yAPC1466 for β-catenin down-regulation, since the construct was almost inactivated upon the introduction of point mutations that abolish β-catenin binding to the 15R (**figure 4C**) [17]. In contrast, yAPCL1241 truncated at the equivalent position, but lacking naturally the 15R region, was fully competent in inhibiting the transcriptional activity of β-catenin (**figure 4B**). Thus, truncated yAPC containing the CiD requires the 15R region to inhibit β-catenin whereas yAPCL does not.

### Phosphorylated β-catenin Binds Preferentially to the 15R than to the 20R1 of APC

**Figure 5:**
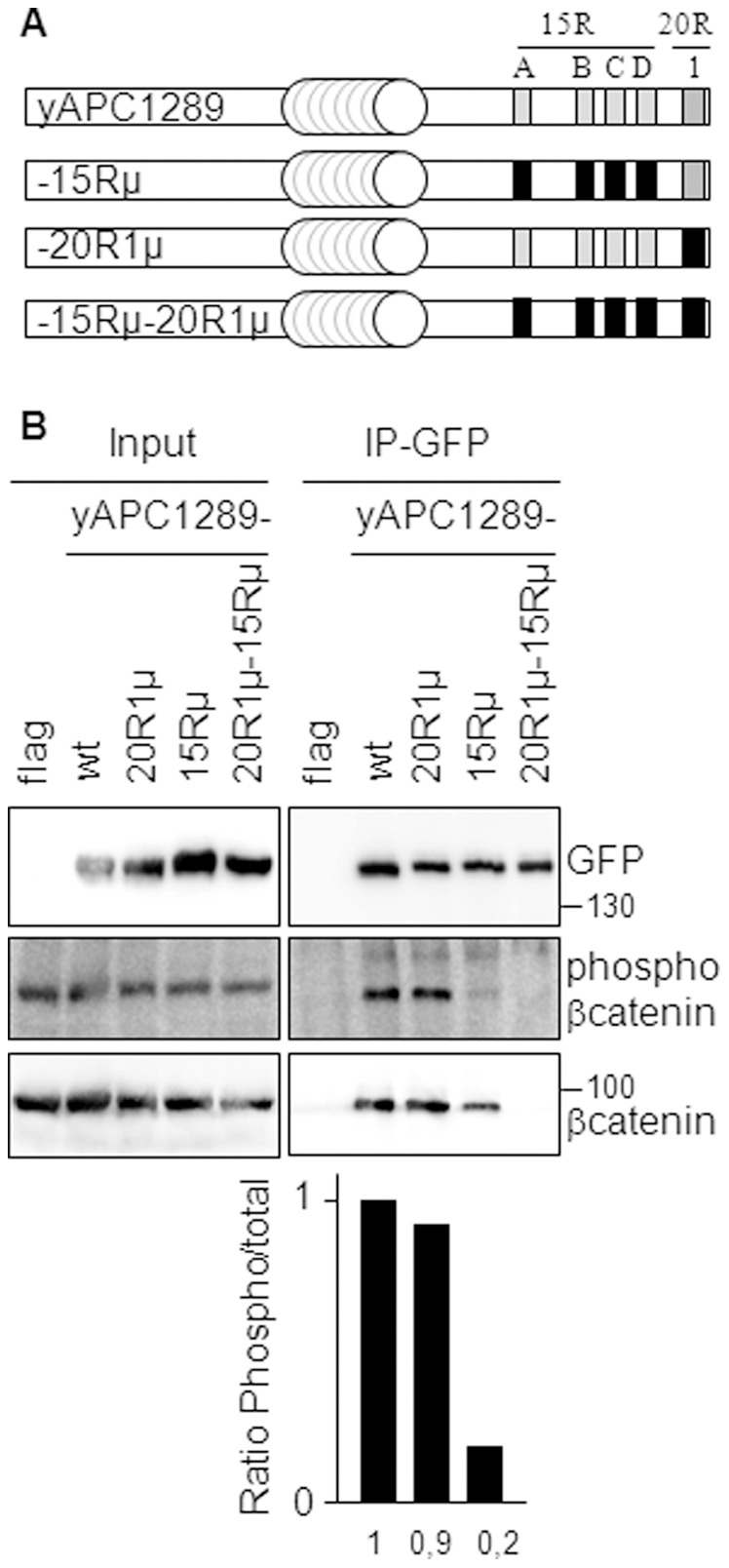
Phosphorylated β-catenin binds preferentially to the 15R rather than to the 20R1 of APC. **A**, Schematic representation of yAPC1289 and mutants thereof. See figure 1 for the nomenclature. The repeats indicated in black are mutated and cannot bind to β-catenin. **B**, 293T cells were transiently transfected on day 1 with either an empty plasmid (flag) or expression vectors for the indicated APC constructs tagged with YFP at their N-terminus. Triton-X100 cell extracts containing 50 mM NaF were prepared on day 3, mixed with a NaF supplemented Triton-X100 cell extract from SW480 as a source of β-catenin and subjected to immunoprecipitation using an anti-GFP antibody (IP-GFP). Pulled-down proteins were visualized by western blotting analysis, using the indicated antibodies. Molecular weights are shown in kD. The diagram below shows the ratio of phosphorylated β-catenin to total β-catenin, obtained after densitomeric analysis of the signals revealed by the β-catenin antibodies in the immunoprecipitates.

To understand the role of the 15Rs, we compared binding of phosphorylated β-catenin to the 15R and the 20R1 in immunoprecipitation experiments (**figure 5**). We used yAPC constructs truncated at position 1289, with various combinations of mutant 20R1 and/or 15Rs containing each several amino acid substitutions known to abolish β-catenin binding (**figure 5A**) [17]. These constructs were tested for their ability to immunoprecipitate either phosphorylated β-catenin (Pβ-catenin) or total β-catenin (**figure 5B**). The experiment confirmed the better binding ability of total β-catenin for the 15Rs as compared to the 20R1 [19] since inactivating mutations in the 15R (as in yAPC1289-15µ) led to a stronger reduction of β-catenin binding than those in the 20R1 (as in yAPC1289-20R1µ). When we analyzed the amount of Pβ-catenin pulled down by yAPC1289-15Rµ, we found that the reduction in binding provoked by mutation of the 15Rs was more pronounced for Pβ-catenin than for total β-catenin (approximately 5-fold). We did not observe this difference between Pβ-catenin and total β-catenin upon mutation of the 20R1. We concluded that Pβ-catenin binds preferentially to the 15Rs than to the 20R1. Consequently, this result suggested that the 15Rs constitute a gate connecting phosphorylation of β-catenin to the subsequent transfer of phospho-β-catenin to the ubiquitination machinery. By extension, it suggested also that APCL may use the 15Rs of APC to catalyze β-catenin degradation, since the extent of homology between APC and APCL, besides the presence/absence of the 15Rs, makes it difficult to consider two radically different mechanisms of action.

### APCL Depends on APC to Inhibit the Transcriptional Activity of β-catenin

**Figure 6:**
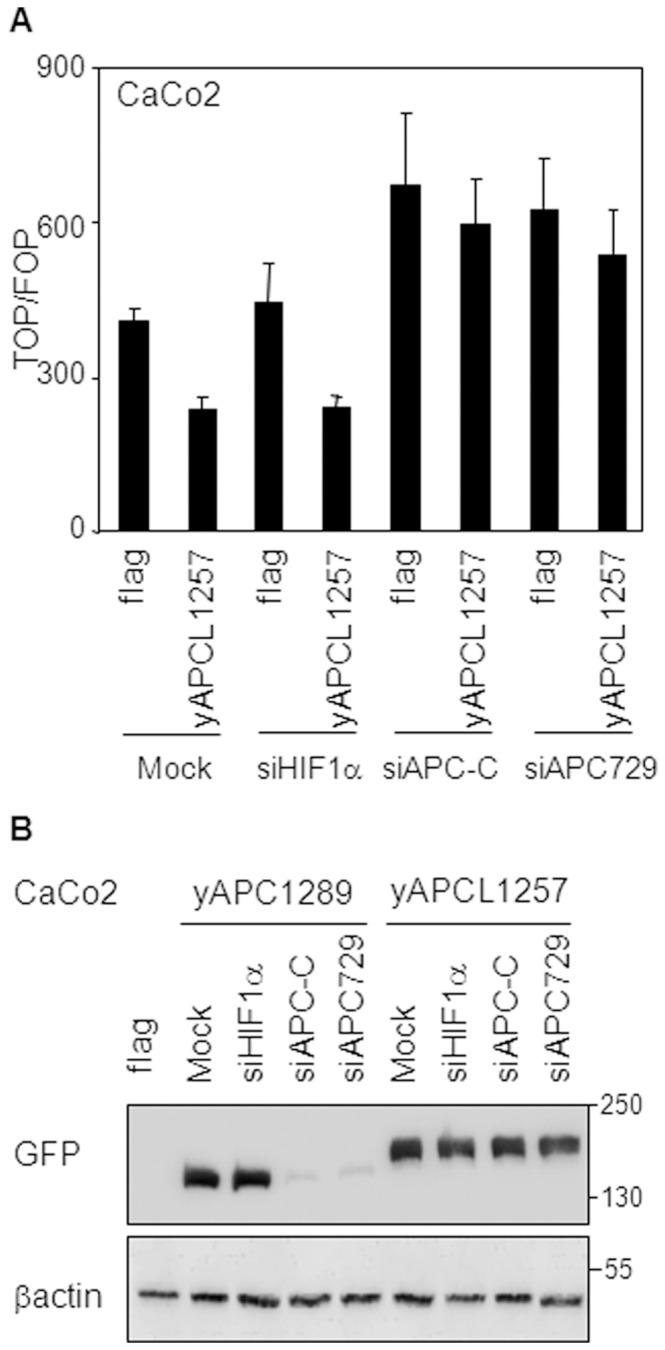
The inhibition of the transcriptional activity of β-catenin mediated by APCL depends on truncated APC. **A**, CaCo2 cells were transiently transfected on day 1 with the indicated siRNAs, on day 2 with reporter plasmids together with 30 ng of either an empty plasmid or an expression vector for the APCL construct yAPCL1257 (figure 1) and subjected on day 3 to the TOP/FOP reporter assay to measure the transcriptional activity of β-catenin (see Methods). Shown is the mean of three independent values +/− standard deviation from a representative experiment. **B**, CaCo2 cells were transiently transfected on day 1 with the indicated siRNAs and on day 2 with either 2 µg of an empty plasmid (flag) or expression vectors for either truncated APC (yAPCL1289, 2 µg) or APCL (yAPCL1257, 0,5 µg) (figure 1). Whole cell extracts were prepared on day 3 and subjected to western blotting analysis, using the indicated antibodies. Molecular weights are shown in kD. Note that yAPCL1257 is running slower than yAPCL1289.

To investigate the possibility of a functional connection between APCL and APC, we compared, using the TOP/FOP transcriptional assay, the β-catenin down-regulating activities of yAPCL1257 in the presence of truncated APC from CaCo2 cells, and after reduction of the latter’s endogenous levels by RNA interference. Ectopic expression of yAPCL1257 either in the absence of siRNA or after transient expression of a siRNA against HIF1α as a negative control led to an inhibition of β-catenin transcriptional activity (**figure 6A**). Transient transfection of two different siRNAs against APC enhanced the basal level (**figure 6A**). Meanwhile, the efficiency of yAPCL1257 was substantially reduced in the presence of either siRNA against APC. Control experiments revealed that the siRNAs against APC were not altering the expression level of yAPCL1257 (**figure 6B**). In SW480 cells, the activity of yAPCL1257 was not influenced by down-regulation of APC (unpublished data). This might be linked to the difficulty of down-regulating efficiently endogenous APC to a limiting level, as previously discussed [33]. We concluded that APC complements the activity of APCL in inhibiting the transcriptional activity of β-catenin in CaCo2 cells.

### The 15Rs of APC Facilitate APCL-dependent Regulation of β-catenin Level and Activity Specifically

**Figure 7:**
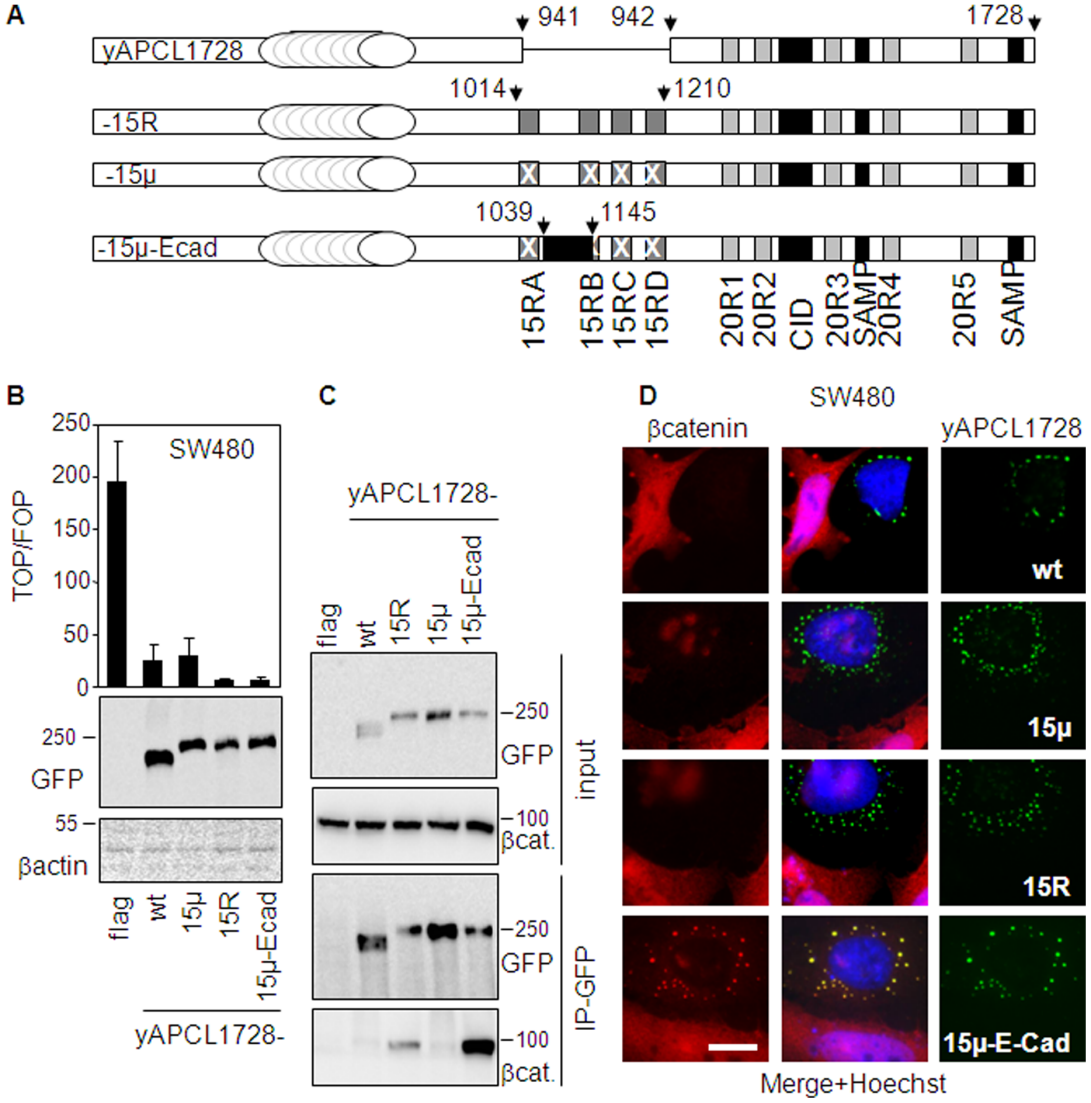
The transfer of the 15R region of APC in APCL enhances the inhibition of the β-catenin transcriptional activity. **A**, Schematic representation of APCL chimaeric constructs truncated at position 1728 and tagged with YFP at the N-terminus (see figure 1 for the nomenclature). yAPCL1728-15R contains the 15 amino acid repeat region of APC (residues 1014–1210) inserted between residues 941 and 942 of APCL. yAPCL1728-15µ contains three point mutations in each 15R that abolish β-catenin binding. yAPCL1728-15µ-Ecad contains the mouse Ecadherin β-catenin binding site (residues 629–728) inserted between residues 1039 and 1145 of APC. **B**, SW480 cells were transiently transfected on day 1 with reporter plasmids, together with 30 ng of either an empty vector (flag) or expression vectors for the indicated APCL constructs. TOP/FOP reporter assays were performed on day 3 to measure the transcriptional activity of β-catenin (see Material and Methods). Shown is the mean of three independent values +/− standard deviation from a representative experiment. In parallel, cells were transiently transfected on day 1 with 1 µg of the indicated constructs and whole cell extracts were prepared on day 3 and subjected to western blotting analysis using the indicated antibodies. Molecular weights are shown in kD. **C**, 293T cells were transiently transfected on day 1 with either a control vector (flag) or expression plasmids for the indicated APCL constructs. Triton-X100 cell extracts were prepared on day 3, mixed with an SW480 Triton-X100 extract as a source of β-catenin and subjected to immunoprecipitation using an anti-GFP antibody (IP-GFP). Pulled-down proteins were revealed by western blotting analysis using the indicated antibodies. **D**, SW480 cells were transiently transfected on day 1 with 1 µg of the indicated APCL constructs. Cells were fixed on day 3 and stained with an antibody against β-catenin and the Hoechst dye. The green, red and blue colours correspond to the fluorescences associated with YFP, β-catenin and the Hoechst dye, respectively.Bar, 10 µm.

To further explore this functional association, we incorporated the 15R region (amino acid residues 1014 to 1210) of APC into yAPCL1728 (between amino acid residue 941 and 942, yAPCL1728-15R) (**figure 7A**). We also constructed a modified version of yAPCL1728-15R, containing several point mutations in each 15R and known to affect β-catenin binding and the activity of truncated APC (yAPCL1728-15µ). This was done as a control to evaluate any influence of topological alterations on the activity of yAPCL1728. In addition, we also created a third construct by replacing the second mutant 15R from yAPCL1728-15µ by the β-catenin binding site from mouse E-cadherin which is also known to bind phosphorylated β-catenin (yAPCL1728-15µ-Ecad) [35]. The three constructs yAPC1728-15R, yAPCL1728-15µ and yAPCL1728-15µ-Ecad were transiently expressed in SW480 cells and compared to yAPCL1728 in their ability to inhibit the transcriptional activity of β-catenin (**figure 7B**). We found that the mutated 15R region did not modify the inhibitory activity of yAPCL1728 toward β-catenin but the wild-type 15Rs as well as the β-catenin binding site of E-cadherin enhanced it substantially. Immunoprecipitation experiments revealed that the 15Rs region conferred a detectable β-catenin binding activity to yAPCL1728 and that the β-catenin binding site of E-cadherin was binding better to β-catenin than the 15R region (**figure 7C**), as previously described [36]. Immunofluorescence analysis showed that all constructs had a similar if not identical intracellular localization (**figure 7D**). However, and in contrast to the other constructs, yAPCL1728-15µ-ECad was retaining part of the β-catenin pool instead of down-regulating it. This retention is possibly due to the high affinity for β-catenin attributed to the E-cadherin fragment [36] and likely explains the increased inhibitory activity of yAPCL1728-15 µEcad toward β-catenin mediated transcription by a sequestration effect. Thus, a higher affinity for β-catenin does not confer per se a better down-regulation of β-catenin level. We concluded that the 15Rs facilitate APCL-dependent regulation of β-catenin level and activity specifically.

### APC-APCL Hetero-complexes are Different from APC-APC Homo-complexes

APCL may benefit from the 15Rs of APC through heterodimerization and it has been reported previously that under endogenous settings an antibody against APCL can co-immunoprecipitate the SW480 APC isoform truncated at position 1338, typical of colon cancer cells [26]. APC truncations occurring before the CiD lead to the formation of dots or homo-complexes that are likely the consequences of oligomerization occurring through both dimerization domains bracketing the armadillo repeat (**figure 1**) [20]. This property of forming dots helped visualizing colocalisation of truncated APC with APCL, thereby providing an indication that the two proteins have the opportunity to interact also with each other in DLD1 cells (**figure S3**). This is in line with the results of the immunoprecipitation experiment mentioned above and previously performed in SW480 cells [26]. Note that the fiber-like appearance of yAPCL was predominant over the dotty pattern in DLD1 cells (**figure S3**), in contrast to the situation observed in SW480 cells (**figure S1**).

**Figure 8:**
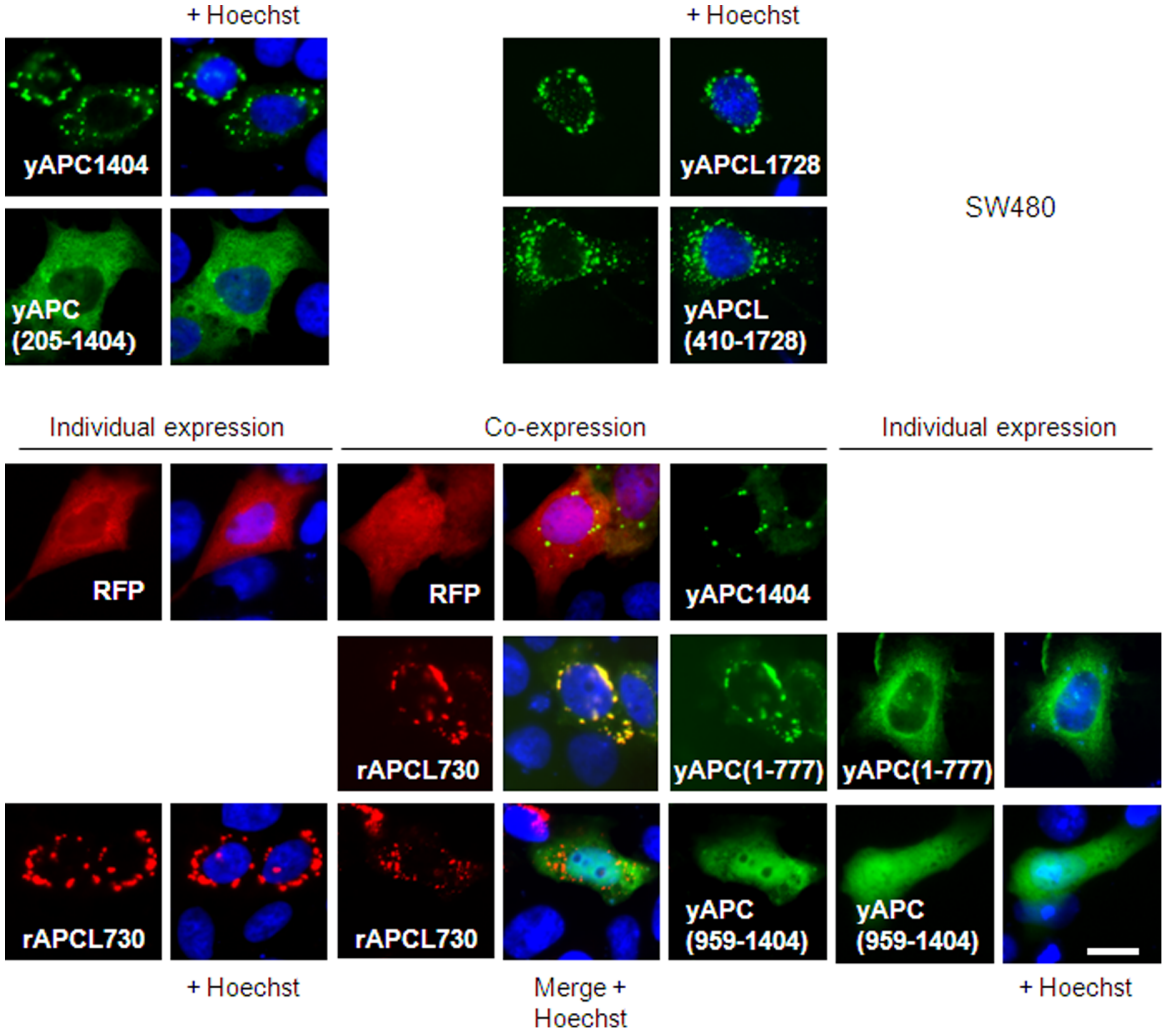
**APC homo-complexes are different from APCL homo-complexes and APC-APCL hetero-complexes.** SW480 cells were transiently transfected on day 1 with 1 µg of either RFP, the indicated APC and APCL constructs tagged with YFP at their N-terminus (see figure 1) or the APCL construct rAPCL730 tagged with RFP at the N-terminus, either alone or in the indicated combinations. Cells were fixed on day 3 and stained with the Hoechst dye. The green, red and blue colours correspond to the fluorescences of YFP, RFP and the Hoechst dye, respectively. Bar, 10 µm.

To understand the discrepancy between the active yAPCL1241 and the inactive yAPC1466-15µ that are both supposed to interact with endogenous truncated APC, we compared the structural requirements leading to APC homo-complexes and APC-APCL hetero-complexes in SW480 (**figure 8**) and DLD1 cells (**figure S4**). The APC dots disappeared with the removal of the first dimerization domain (compare yAPC1404 and yAPC(205-1404)). In contrast, an even larger N-terminal deletion in truncated APCL still resulted in the retention of the dotty pattern (compare yAPCL1728 and yAPCL(410-1728). When we compared the localizations of yAPC777 lacking the second dimerization domain and rAPCL730 (tagged with the red fluorescent protein (RFP) at the N-terminus) which are almost of equivalent lengths, we found that the former one was diffusely distributed, whereas the latter one kept the dotty pattern. Thus, APC and APCL dots are of a different nature, which is in agreement with the reported cytoplasmic oligomerization of truncated APC [20] and Golgi localization of APCL [26]. Importantly, co-expression resulted in the recruitment of yAPC777 in rAPCL730 dots. Appropriate controls using RFP and an unrelated APC fragment yAPC(958-1404) excluded the involvement of the YFP and RFP moieties in this recruitment. We concluded that the formation of APC-APCL hetero-complexes is submitted to a different set of structural rules than those underlying the formation of APC-APC homo-complexes. This provided a plausible explanation for the difference of activity observed between yAPCL1241 and yAPC1466-15µ in inhibiting β-catenin (**figure 4**).

## Discussion

Our experiments show for the first time that colon cancer cells express a functional APCL (or APC2) that controls the level and/or the transcriptional activity of β-catenin. They suggest that APCL uses the 15R region of APC to target β-catenin for degradation and offer a plausible explanation as to why APCL is not mutated and why the truncating mutations in APC almost always lead to the retention of a fragment of minimal length in colon tumour cells.

### APCL is not Efficient

Ectopic expression experiments revealed that APCL is less efficient than APC at inhibiting the transcriptional activity of β-catenin. This is linked to a weaker ability to bind β-catenin, as observed in co-immunoprecipitation experiments (**figure 3** **)**. Thus, colon cancer cells expressing truncated APC may tolerate a wild-type APCL because it displays a leaky activity, allowing β-catenin to escape degradation in a sufficient amount to initiate tumourigenesis.

### APC Sustains the Activity of APCL

Ectopic expression of APCL truncated just after the CiD (the minimal length for either APC or APCL required for β-catenin down-regulation, **figure 4**) was accompanied by down-regulation of the level of β-catenin and inhibition of its transcriptional activity. This result was surprising because APCL lacks the 15Rs region and an APC construct truncated at an equivalent position is inactive when the 15Rs are mutated to abolish any possibility of β-catenin interaction [17], [19]. Thus, APCL does not require the 15Rs to down-regulate the β-catenin level, whereas APC does.

Beside the absence of the 15R region, the relatively high conservation in APCL of the APC domains known to be important for β-catenin degradation makes it difficult to consider two different mechanisms of action. Functionally, this is illustrated by the observation that both APC and APCL require the same CiD domain to down-regulate the level of β-catenin and inhibit its transcriptional activity (**figure 4**). Therefore, the apparent functional similarity leads to the possibility that APCL may rely on the 15R of APC for β-catenin degradation. Previous reports and our current results are in line with this hypothesis. First, the activity of ectopically expressed APCL decreases upon reduction of the endogenous level of APC by RNA interference (**figure 6**). This result corroborates data indicating that the activity of E-APC can depend on D-APC (the drosophila orthologs of APCL and APC, respectively) [29], [37]. Second, the introduction of the 15Rs region of APC in APCL enhances the latter’s inhibitory activity toward the transcriptional activity of β-catenin (**figure 7**). This effect is specific of the 15Rs, because the introduction of the E-cadherin β-catenin binding site in APCL led rather to an inhibition of the down-regulation of β-catenin level. Third, previous reports have shown that APC stimulates β-catenin phosphorylation [38], [39]. More specifically, this activity was associated with an internal APC fragment extending from the end of the 20R1 to the end of the 20R3. The fact that this fragment is highly conserved in APCL suggests that both APC and APCL can stimulate β-catenin phosphorylation. Meanwhile, our data show that phosphorylated β-catenin displays a preference for binding to the 15Rs rather than to the 20R1 of APC (**figure 5**). This observation suggests that the 15Rs represent a gate linking β-catenin phosphorylation to its subsequent ubiquitination/degradation. Thus, perhaps APCL may perform only the early but not the late steps leading to β-catenin degradation due to the absence of the 15R region. Fourth, truncated APC and APCL can heterodimerize. This has been demonstrated previously with the endogenous proteins [26] and sustained here using ectopic expression associated with immunofluorescence detection (**figure S3**). Heterodimerization might be important for activity by allowing a direct transfer of phosphorylated β-catenin onto the 15Rs. Accordingly, a transient release of phosphorylated β-catenin in the cytoplasm is unlikely because the ectopically expressed APC construct inactivated by the introduction of point mutations that prevent β-catenin binding to the 15R (**figure 4**) would also be complemented by endogenous truncated APC. In addition, there is evidence that phosphorylated β-catenin is particularly sensitive to the action of a phosphatase in the absence of a protection mechanism provided by APC [40]. Recently, a model for the degradation of β-catenin has been proposed [37], involving hetero-oligomerization of D-APC and E-APC that may act cooperatively in the destruction complex. Our current work hypothesis is similar and includes the notion that APCL from colon cancer cells may catalyze the phosphorylation of β-catenin to some extent, but the subsequent processing of phosphorylated β-catenin may occur on the 15R provided by truncated APC.

Both APC and APCL can form individually higher-order structures visualized as cytoplasmic dots. This clustering has also been described for drosophila E-APC and occurs through self-association of the armadillo repeats [24]. This is contrasting with the rules of APC homo-oligomerization that involve the dimerization domains located on both sides of the armadillo repeat domain [20], [23], [25]. The results presented here also illustrate this variation. Accordingly, APC truncated shortly before or after the armadillo repeat domain cannot build dots anymore, whereas APCL still retains this capacity after similar deletions (**figure 8**). Thus, truncated APC homo-complexes are of a different nature than APCL homo-complexes, implying different functions. Further, an APC mutant that cannot form dots anymore is recruited in the complexes built by an APCL mutant of equivalent length (**figure 8**). It has also been shown that the armadillo repeat domains of mammalian APCs can hetero-dimerize [27]. Thus, APC homo-complexes are also of a different nature than APC-APCL hetero-complexes. Previous data suggest that truncated APC homo-oligomers can actually function as inhibitors of β-catenin degradation [28]. The two different types of complexes may explain why endogenous truncated APC can complement the activity of APCL but cannot rescue the fatal consequences of inactivating the 15R in a truncated APC construct retaining the CiD (**figure 4**).

The evidence of an APCL activity in colon cancer cells (**figure 2**) combined with the observation that it depends on APC (**figure 6**) may provide an explanation for the following couple of previously published paradoxical observations. When APC truncated before the CiD is ectopically expressed in colon cancer cells it never down-regulates the level of β-catenin [28], [34]. In contrast, when the endogenous counterpart of a similar length and therefore also lacking the CiD is down-regulated by RNA interference, the level of β-catenin increases, at least in some cell lines [33]. This indicates that an activity down-regulating the β-catenin level remains associated with truncated APC despite the absence of the CiD. Thus, in light of our current findings, it appears that the removal of endogenous truncated APC leads to enhanced stabilization of β-catenin possibly due to a loss of the 15Rs function. Exogenous truncated APC does not affect the β-catenin level because APCL may remain the limiting component of the system.

### APCL and the Selection of APC Mutations in Colorectal Cancer

The APC dependence of APCL suggests that colon cancer cells must maintain some control over the level of β-catenin. This is known as the “just right signalling” hypothesis stating that truncating mutations of APC are selected to avoid “too much” β-catenin signalling that would be detrimental for cell proliferation [41]–[43]. The analysis of tumours from FAP patients has revealed that cancer cells always retain a truncated APC which very rarely lacks the first 15R (2 cases out of 328 tumours) [19], [20]. In view of our results, it is tempting to recognize in this selection the need to confer a β-catenin down-regulating activity to APCL by the retention of at least one 15R in truncated APC. Thus, the selection of APC truncating mutations in colon cancer may relate to the dependence of APCL for APC.

## Materials and Methods

### Cells

Human embryonic kidney cells expressing the SV40 large T antigen (HEK293T), HeLa cells and CaCo2, SW837, LoVo, HT29, SW480 and DLD1 colorectal cancer cell lines were all obtained from the American Type Culture Collection (ATCC) and maintained in DMEM medium (PAA Laboratories, Cölbe, Germany) supplemented with 10% fetal calf serum (Perbio Laboratories, Frankfurt/Main, Germany) and 1% penicillin and 1% streptomycin (PAA Laboratories). APC mutations in the cell lines are described in [44] and the resulting truncated APC protein products are shown in [33].

### Antibodies

The mouse anti-APCL antibody was purchased from Biozol (Eching, Germany). Secondary antibodies coupled to either horseradish peroxidase or Cy3 were from Dianova (Hamburg, Germany), anti-GFP from Roche (Mannheim, Germany), anti-APC antibody Ali(12–28) from Abcam (Cambridge, UK), anti-β-actin and anti-lamin from Santa Cruz Biotechnologies (Heidelberg, Germany), anti-α-tubulin from AbD Serotec (Düsseldorf, Germany), rabbit anti-flag, anti-E-cadherin and anti-β-catenin from Sigma (Taufkirchen, Germany) and anti-phospho-β-catenin (ser33/37) from Pierce Biotechnology (Rockford, USA).

### Plasmids

The plasmids expressing the YFP-APC, YFP-APCL and RFP-APCL fusion proteins were constructed by standard molecular biology methods. The sequence of any of these plasmids is available upon request. pcDNAflag [30] was used as a control vector. Human APCL was obtained from H. Nakagawa and Y. Nakamura [21], mouse E-cadherin from M. Sachs [45] and mouse N-terminally tagged flag-APC1287 from R. Fodde.

### Plasmid Transfection

Plasmids were transfected into cells overnight using 5 µl polyethylenimine (1 mg/ml) per µg DNA. For transient transfection of plasmids in DLD1 or SW480 cells, 2 µg total DNA/250000 cells/35 mm dish were used. For transient transfection of plasmids in HEK293T cells, 10 µg total DNA/5000000 cells/85 mm dish were used.

### siRNA Transfection

siAPC-C (gacgttgcgagaagttgga), siAPC729 (gaggtcatctcagaacaag), siAPCL413 (ggtgtttcctgctgaatga), siAPCL5100 (ggcgccaattcaattgtca), siHIF1α [46], siHIF2α [46] or siGFP (aagctacctgttccatggcca) (50–100 nM final concentration) were transfected into cells overnight using 1 µl Transit TKO (MoBiTec, Göttingen, Germany) per µl siRNA (20 µM). The sequences show only the coding strand.

### TOP/FOP Reporter Assays [47]

The TOPglow reporter consists of a tandem repeat of four TCF/LEF1 binding sites inserted in front of a TATA box [48], driving the expression of luciferase in a β-catenin-dependent manner. In the FOPglow reporter, the four binding sites are mutated to abolish binding of TCF/LEF1. 300 ng pUHD16.1 plasmid expressing β-galactosidase as an internal control to correct for variations in transfection efficiencies were transiently transfected together with 300 ng of either FOPglow or TOPglow plasmids. The transcriptional activity measured 48h post transfection is defined as the ratio of TOPglow to FOPglow luciferase values corrected by the β-galactosidase values. All assays in this study have been performed at least twice.

### Cell Extracts

Hypotonic cel extracts were prepared in a 25 mM TrisHCl pH7.5 buffer containing 1 mM EDTA, 1 mM DTT and 1 mM PMSF. Triton-X100 extracts were prepared in a 20 mM TrisHCL pH7.4 buffer containing 150 mM NaCl, 5 mM EDTA, 1% Triton-X100, 1 mM DTT and 1 mM PMSF.

### Immunoprecipitation

were performed in Triton-X100 extracts and the immunoprecipitates were washed in a 50 mM TrisHCl pH8 buffer containing 150 mM NaCl, 5 mM EDTA and 1% Triton-X100. Immunoprecipitations combined cell extracts from 293T cells expressing high levels of the different YFP-APC(L) fusions (due to a very high transfection efficiency), mixed with a cell extract from SW480 cells as an abundant source of β-catenin.

### Western Blotting

The blots were developed using the chemiluminescence reagents Western Lightning™ (Perkin Elmer Life Sciences, Boston, MA) and the signals were detected under a LAS-3000-Fuji camera from Raytest (Straubenhardt, Germany).

### Immunofluorescence

was conducted as described [49]. Imaging of Hela cells was done on a Zeiss LSM710 confocal microscope (Zeiss MicroImaging, Jena, Germany) equipped with an Ar-Laser Multiline (458/488/514 nm), a DPSS-561-10 (561 nm), a Laser diode 405-30 CW (405 nm), and a HeNe-laser (633 nm). The objective was a Zeiss plan-Apochromat 63x/1.4-Oil-DIC-III. Image acquisition was done with the software ZEN2010 (Zeiss MicroImaging) and the three-dimensional reconstruction of Z-stacks with Imaris (Version 7.1.1, Bitplane, Zürich, Switzerland).

## Supporting Information

Figure S1 **Comparison of yAPC and yAPCL in down-regulating the level and inhibiting the transcriptional activity of β-catenin.** **A**, APCL abolishes β-catenin accumulation in SW480 cells. SW480 cells were transiently transfected on day 1 with expression vectors for either full length APC or full length APCL tagged with YFP at their N-terminus. Cells were fixed on day 3 and stained with an antibody against β-catenin and the Hoechst dye. The green, red and blue colours correspond to the fluorescences associated with YFP, β-catenin and the Hoechst dye, respectively. Bar, 10 µm. **B**, APCL is less efficient than APC in inhibiting the transcriptional activity of β-catenin. CaCo2 cells were transiently transfected on day 1 with reporter plasmids, together with either 100 ng of either an empty vector (flag) or yAPC or increasing amounts of yAPCL, as indicated. TOP/FOP reporter assays were performed on day 3 to measure the transcriptional activity of β-catenin (see Material and Methods). Shown is the mean of three independent values +/− standard deviation from a representative experiment.
(PDF)

Figure S2 **3D reconstruction of yAPCL1728 localization.** Hela cells were transiently transfected with an expression vector for yAPCL1728. Cells were fixed 24 h after transfection, stained with Hoechst dye and imaged on a confocal microscope. The 3D reconstruction visualizes the localization of yAPCL1728 in cytoplasmic dots. The green and blue colours correspond to the fluorescences associated with YFP and the Hoechst dye, respectively. Bar, 7 µM.
(PDF)

Figure S3 **Truncated APC colocalizes with full length APC and full length APCL.** DLD1 cells were transiently transfected on day 1 with expression vectors for either YFP, APC truncated at position 1289 and flag-tagged at the N-terminus or full length APC and APCL tagged with YFP at their N-terminus, either alone or in the indicated combinations. Cells were fixed on day 3 and stained with an anti-flag antibody and the Hoechst dye. Note that none of the constructs is imposing its own localization to the other one in a dominant manner in the cell population. Similar results were obtained when replacing the flag tag by the red fluorescent protein (data not shown). The green, red and blue colours correspond to the fluorescences associated with YFP, the flag tag and the Hoechst dye, respectively. Bar, 10 µm.
(PDF)

Figure S4 **APC homo-complexes are different from APCL homo-complexes and APC-APCL hetero-complexes.** DLD1 cells were transiently transfected on day 1 with 1 µg of either RFP, the indicated APC and APCL constructs tagged with YFP at their N-terminus (see figure 1) or the APCL construct rAPCL730 tagged with RFP at the N-terminus, either alone or in the indicated combinations. Cells were fixed on day 3 and stained with the Hoechst dye. The green, red and blue colours correspond to the fluorescences of YFP, RFP and the Hoechst dye, respectively. Bar, 10 µm.
(PDF)
