# Supplementary figures and images for: Functional Comparison of Human Adenomatous Polyposis Coli (APC) and APC-Like in Targeting Beta-Catenin for Degradation
Source: PLoS One. 2013 Jul 1;8(7):e68072. doi: 10.1371/journal.pone.0068072 (PMC3698177; doi:10.1371/journal.pone.0068072)

Figure S1

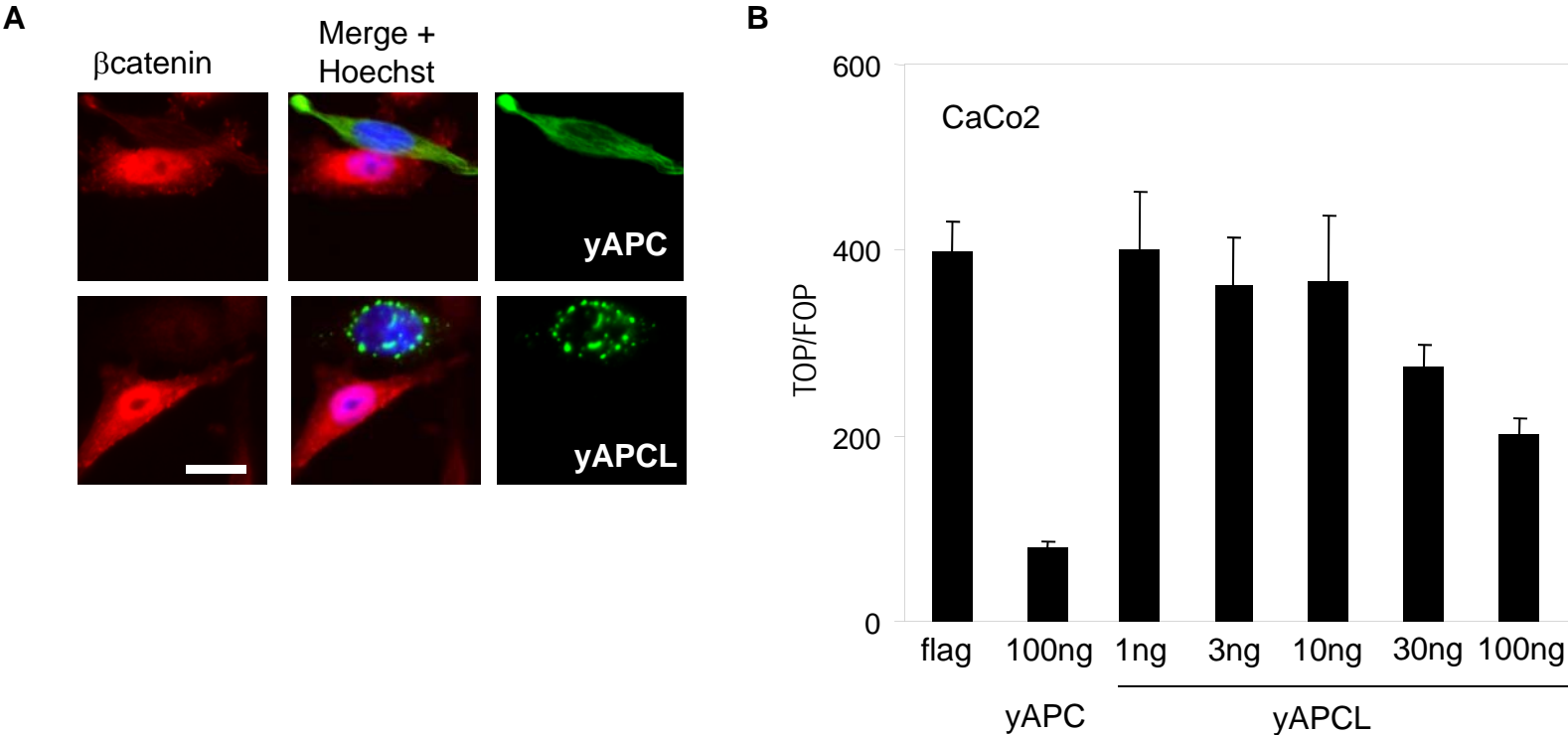

Supplement: Figure S1 — Comparison of yAPC and yAPCL in down-regulating the level and inhibiting the transcriptional activity of β-catenin. A, APCL abolishes β-catenin accumulation in SW480 cells. SW480 cells were transiently transfected on day 1 with expression vectors for either full length APC or full length APCL tagged with YFP at their N-terminus. Cells were fixed on day 3 and stained with an antibody against β-catenin and the Hoechst dye. The green, red and blue colours correspond to the fluorescences associated with YFP, β-catenin and the Hoechst dye, respectively. Bar, 10 µm. B, APCL is less efficient than APC in inhibiting the transcriptional activity of β-catenin. CaCo2 cells were transiently transfected on day 1 with reporter plasmids, together with either 100 ng of either an empty vector (flag) or yAPC or increasing amounts of yAPCL, as indicated. TOP/FOP reporter assays were performed on day 3 to measure the transcriptional activity of β-catenin (see Material and Methods). Shown is the mean of three independent values +/− standard deviation from a representative experiment. (PDF) [file pone.0068072.s001.pdf]

**Figure S2**

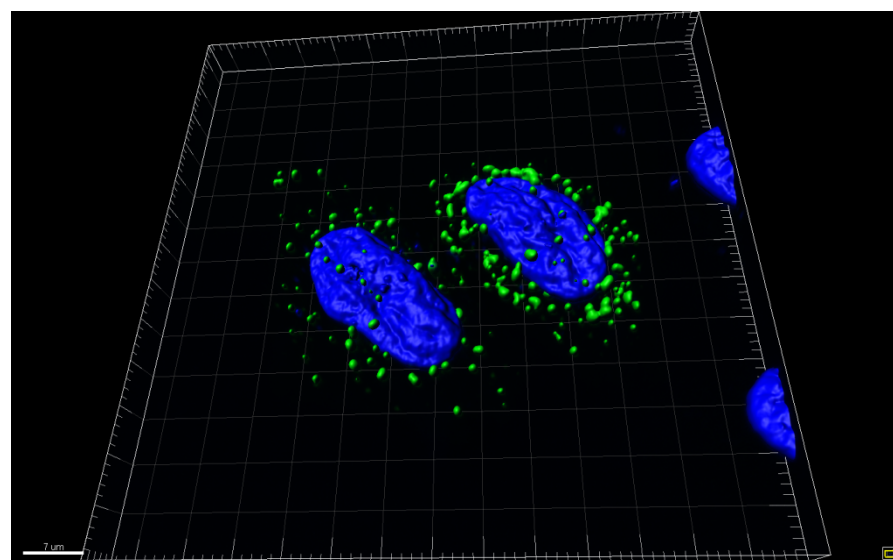

Supplement: Figure S2 — 3D reconstruction of yAPCL1728 localization. Hela cells were transiently transfected with an expression vector for yAPCL1728. Cells were fixed 24 h after transfection, stained with Hoechst dye and imaged on a confocal microscope. The 3D reconstruction visualizes the localization of yAPCL1728 in cytoplasmic dots. The green and blue colours correspond to the fluorescences associated with YFP and the Hoechst dye, respectively. Bar, 7 µM. (PDF) [file pone.0068072.s002.pdf]

**Figure S3**

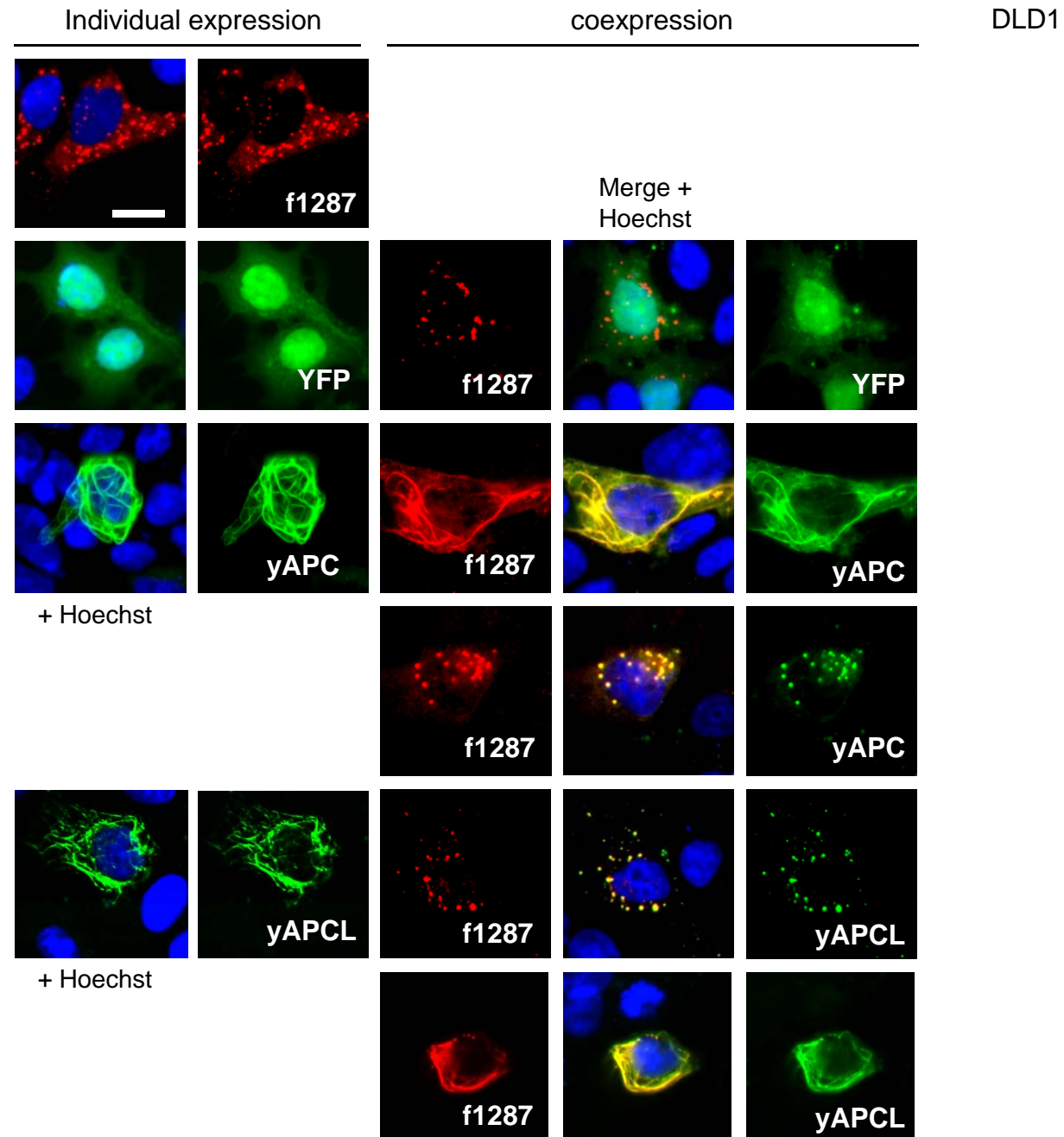

Supplement: Figure S3 — Truncated APC colocalizes with full length APC and full length APCL. DLD1 cells were transiently transfected on day 1 with expression vectors for either YFP, APC truncated at position 1289 and flag-tagged at the N-terminus or full length APC and APCL tagged with YFP at their N-terminus, either alone or in the indicated combinations. Cells were fixed on day 3 and stained with an anti-flag antibody and the Hoechst dye. Note that none of the constructs is imposing its own localization to the other one in a dominant manner in the cell population. Similar results were obtained when replacing the flag tag by the red fluorescent protein (data not shown). The green, red and blue colours correspond to the fluorescences associated with YFP, the flag tag and the Hoechst dye, respectively. Bar, 10 µm. (PDF) [file pone.0068072.s003.pdf]

**Figure S4**

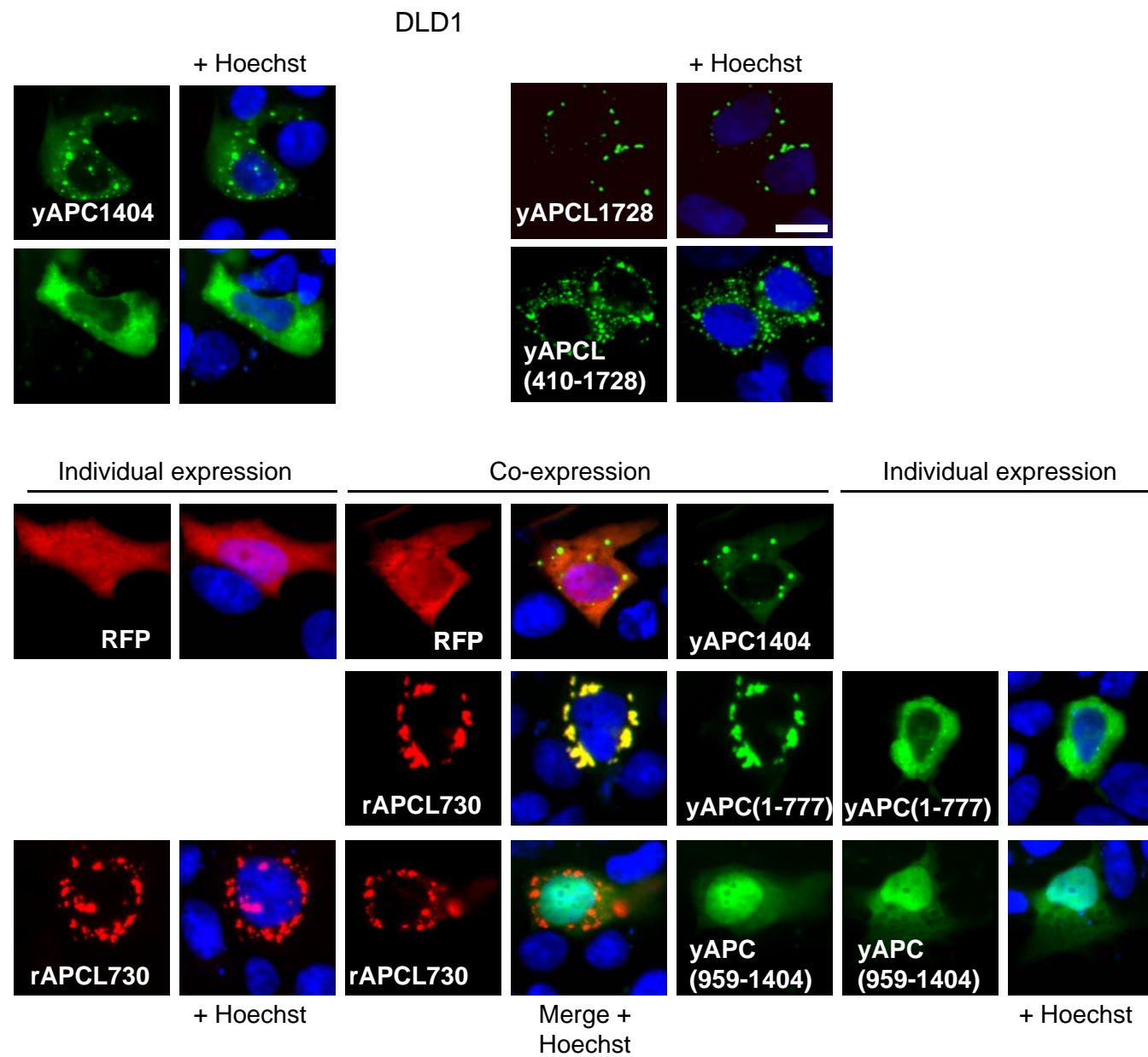

Supplement: Figure S4 — APC homo-complexes are different from APCL homo-complexes and APC-APCL hetero-complexes. DLD1 cells were transiently transfected on day 1 with 1 µg of either RFP, the indicated APC and APCL constructs tagged with YFP at their N-terminus (see figure 1) or the APCL construct rAPCL730 tagged with RFP at the N-terminus, either alone or in the indicated combinations. Cells were fixed on day 3 and stained with the Hoechst dye. The green, red and blue colours correspond to the fluorescences of YFP, RFP and the Hoechst dye, respectively. Bar, 10 µm. (PDF) [file pone.0068072.s004.pdf]
